# Increasing evidence of human infections by the neurotropic Borna disease virus 1 (BoDV-1)

**DOI:** 10.1080/21505594.2023.2218075

**Published:** 2023-06-05

**Authors:** Michaela Cain, Hinh Ly

**Affiliations:** Department of Veterinary & Biomedical Sciences, College of Veterinary Medicine, University of Minnesota, Twin Cities, MN, USA

Human Borna disease virus 1, BoDV-1, is a negative sense, single-stranded, enveloped RNA virus in the family *Bornaviridae* within the order Mononegavirales [2,3]. The viral genome has six known open-reading frames that produce at least six proteins: nucleoprotein (N), phosphoprotein (P), putative matrix protein (M), type 1 membrane glycoprotein (G), and putative viral polymerase (L) [3,4]. The replication and transcription processes of BoDV-1 occur in the host cell’s nucleus and its genome is highly conserved [3]. BoDV-1 has been shown to replicate in cells of the central nervous system, including neurons, astrocytes, and oligodendrocytes. The bicoloured white-toothed shrew is the primary animal reservoir for BoDV-1, which can establish a persistent infection with broad tissue tropism, but without an overt clinical disease [5].

BoDV-1 infection is characterized by immune mediated meningoencephalitis that can often lead to severe complications and death in spillover hosts, such as in horses and sheep [5]. Borna disease in horses has been described since the 18^th^ century, but only in 1885 that it was designated Borna disease following a major horse epidemic in Borna, which is a town in Saxony, Germany [6,7]. It is thought that some livestock can serve as intermediary hosts of BoDV-1; however, zoonotic transmissions of BoDV-1 have been suspected but not definitely confirmed.

BoDV-1 has been found to induce behavioural changes in some infected animals, such as anxiety, aggression, cognitive defects, hyperactivity, and can lead to a form of neurotropic disease that is characterized by T lymphocyte-mediated encephalitis [5]. Other studies, as referenced in a recent article [8], have suggested a potential link between BoDV-1 infection and psychiatric disorders in humans. It is noteworthy that fatal encephalitis caused by BoDV-1 has predominantly been found in regions of Germany, Liechtenstein, Switzerland, and Austria [9] (Figure 1). In contrast, multiple studies cited in a recent article [6] have found BoDV-1 infected people in different regions throughout the world including, the Middle East, China, and Japan, and central Europe, who appear to be healthy or are asymptomatic carriers. For example, 10% of blood donors in southwest China and 30–40% in the Czech Republic have been associated with subclinical BoDV-1 infections [6].

**Figure 1.**
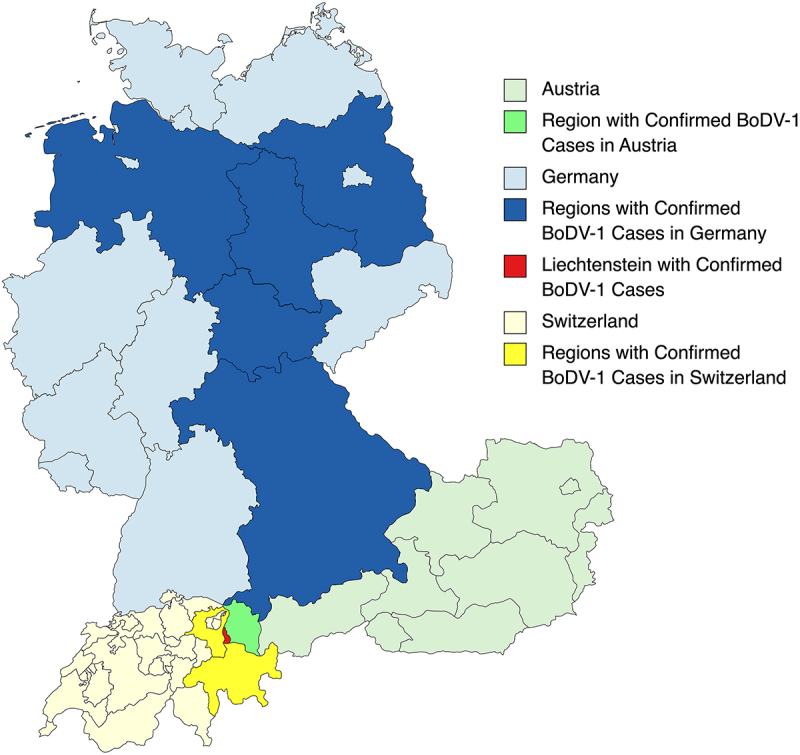
Regions with confirmed human encephalitis cases of Borna disease caused by Borna disease virus 1 (BoDV-1).

The first suspected human cases of Borna disease encephalitis were reported in 1980s. Since then, it has been theorized that a substantial proportion of unidentified human fatal encephalitis cases are caused by BoDV-1, but due to difficulties in developing and validating a test for diagnosing BoDV-1 infection, human cases of BoDV-1 associated encephalitis have not been definitively confirmed [9]. Frank and colleagues have successfully developed and validated a new workflow for rapid testing of BoDV-1 infections using serum and cerebrospinal fluid from at risk patients [1]. The serological workflow uses an indirect immunofluorescence assay followed by a line blot assay, and utilizes the BoDV-1 phosphoprotein (P) antigen. In addition, qRT-PCR and next-generation sequencing were conducted on some patients, who tested positive serologically for BoDV-1 infection. The authors also conducted histopathological characterization of positively confirmed BoDV-1 post-mortem cases. Using these methods, they were able to recover the full-length BoDV-1 genome from the patient’s brain tissue, and upon sequencing the viral genome, they were able to phylogenetically match the viral sequences to BoDV-1 strains found in shrews and domesticated animals of cluster 4 in central Germany [10].

Another study by Liesche and colleagues identified six cases of BoDV-1 infection in six females (17–65 years old) from 1999 to 2019, in brain tissue of encephalitis cases isolated in Bavaria, Germany [11]. All patients developed headache, fever, confusion, deep comas, and died within 2 months of symptom onset (Table 1). In addition, Niller and colleagues reported three previously known cases of encephalitis caused by BoDV-1 in solid-organ transplant, two of which were fatal [5]. Another study done in Germany from 2018 to 2020 examined 103 encephalitis cases of unknown aetiology using qRT-PCR on CSF and brain tissues and found 3% prevalence of BoDV-1 infections [12]. All patients were from Bavaria, who developed encephalitis and fevers, and died within a month of the onset of symptoms. Although more studies need to be done, these recent reported cases suggest an increased risk of BoDV-1 infections in Germany and the potential for severe outcomes in patients who contract the virus.

**Table 1.** Some known human encephalitis cases of Borna disease caused by Borna disease virus 1 (BoDV-1).

Patient Age	Patient Gender	Location	Profession	Time	Symptoms	Outcome	Reference
55	Female	Bavaria	Part-time cleaner	Mid- January 2019	encephalitis, fever, headache, and coma	Death 3 weeks after onset of symptoms	11
11	Female	rural Bavaria	unknown	November 2019	encephalitis, fever, headache, and epileptic seizures	Death 4 weeks after onset of illness	11
79	Male	rural Bavaria	Farmer	June 2020	encephalitis, fever, and confusion	Death 4 weeks after the onset of symptoms	11
74	Female	Bavaria	*	*	Axonal motor neuropathy with Guillain-Barré syndrome-like spread	Death 14 weeks after onset of symptoms	10
21	Female	Bavaria	*	*	Fever, memory deficits, epileptic seizures, progressive loss of consciousness	Death 5 weeks after onset of symptoms	10
13	Female	Bavaria	*	*	Fever, Slurred speech, progressive loss of consciousness	Death 4 weeks after onset of symptoms	10
17	Female	Bavaria	*	*	Fever, headache, confusion, progressive loss of consciousness	Death 6 weeks after onset of symptoms	10
78	Female	Bavaria	*	*	Right-sided weakness, epileptic seizures, progressive loss of consciousness	Death 4 weeks after onset of symptoms	10
55	Female	Bavaria	*	*	Fever, headache, amnesic aphasia, progressive loss of consciousness	Death 2 weeks after onset of symptoms	10

Note: *Not all patient data was available.

Interestingly, people who lived with and had been in close contact with infected patients neither showed signs of disease nor did they harbour BoDV-1 antibodies, which were tested serologically through fluorescence antibody tests and line blots [9]. The only confirmed human-to-human transmission of BoDV-1 was through solid organ transplantation, and it is theorized that all other human cases are spillover events from BoDV-1 infected animals. It has been hypothesized that each human case represents an independent zoonotic transmission event. However, the presence of asymptomatic BoDV-1 carriers in different parts of the world argues for a potential direct transmission of the virus between humans.

There are significant gaps in knowledge about this virus, e.g. how it transmits within and between animal species (intraspecies and interspecies transmissions), and how it can cause disease (disease pathogenesis and pathology), etc. Although the incidence of Borna disease encephalitis seems to be relatively low and is localized to some endemic regions in the world, it is important to conduct routine serological surveys of the virus and to study the disease that it causes that can lead to very high and rapid mortality rate. In addition, its potential link to psychiatric disorders and its increasing geographic presence emphasize the need to study this virus further. Using new molecular tools, such as the reverse genetics system for BoDV-1 [2], researchers have started to make some inroads into understanding the basic biology of this virus. However, until more epidemiological, pathological, virological, and immunological studies are done on BoDV-1 and the disease that it causes in humans, no prophylactic and therapeutic modalities can be developed to prevent or treat these emerging and fatal human viral infections.

## Data Availability

No primary/research data are included in this article.
